# Biochemical and Kinetic Characterization of the Glucose-6-Phosphate Dehydrogenase from *Helicobacter pylori* Strain 29CaP

**DOI:** 10.3390/microorganisms10071359

**Published:** 2022-07-06

**Authors:** Paulina Ortiz-Ramírez, Beatriz Hernández-Ochoa, Daniel Ortega-Cuellar, Abigail González-Valdez, Víctor Martínez-Rosas, Laura Morales-Luna, Roberto Arreguin-Espinosa, Rosa Angélica Castillo-Rodríguez, Luis Miguel Canseco-Ávila, Noemi Cárdenas-Rodríguez, Verónica Pérez de la Cruz, Alba Mónica Montiel-González, Fernando Gómez-Chávez, Saúl Gómez-Manzo

**Affiliations:** 1 Laboratorio de Bioquímica Genética, Instituto Nacional de Pediatría, Secretaría de Salud, Mexico City 04530, Mexico; paulo.r1396@gmail.com (P.O.-R.); ing_vicmr@hotmail.com (V.M.-R.); lauraeloisamorales@ciencias.unam.mx (L.M.-L.); 2 Laboratorio de Inmunoquímica, Hospital Infantil de México Federico Gómez, Secretaría de Salud, Mexico City 06720, Mexico; beatrizhb_16@comunidad.unam.mx; 3 Programa de Posgrado en Biomedicina y Biotecnología Molecular, Escuela Nacional de Ciencias Biológicas, Instituto Politécnico Nacional, Mexico City 11340, Mexico; 4 Laboratorio de Nutrición Experimental, Instituto Nacional de Pediatría, Secretaría de Salud, Mexico City 04530, Mexico; dortegadan@gmail.com; 5 Departamento de Biología Molecular y Biotecnología, Instituto de Investigaciones Biomédicas, Universidad Nacional Autónoma de México, Mexico City 04510, Mexico; abigaila@biomedicas.unam.mx; 6 Posgrado en Ciencias Biológicas, Universidad Nacional Autónoma de México, Mexico City 04510, Mexico; 7 Departamento de Química de Biomacromoléculas, Instituto de Química, Universidad Nacional Autónoma de México, Mexico City 04510, Mexico; arrespin@unam.mx; 8 Programa Investigadoras e Investigadores por México, CONACYT-Instituto Nacional de Pediatría, Secretaría de Salud, Mexico City 04530, Mexico; racastilloro@conacyt.mx; 9 Facultad de Ciencias Químicas, Campus IV, Universidad Autónoma de Chiapas, Tapachula City 30580, Mexico; cansecoavila@gmail.com; 10 Laboratorio de Neurociencias, Instituto Nacional de Pediatría, Secretaría de Salud, Mexico City 04530, Mexico; noemicr2001@yahoo.com.mx; 11 Neurobiochemistry and Behavior Laboratory, National Institute of Neurology and Neurosurgery “Manuel Velasco Suárez”, Mexico City 14269, Mexico; veped@yahoo.com.mx; 12 Centro de Investigación en Genética y Ambiente, Universidad Autónoma de Tlaxcala, Aut. San Martín Texmelucan-Tlaxcala Km 10.5, San Felipe Ixtacuixtla, Tlaxcala 90120, Mexico; amonicamg@yahoo.com; 13 Laboratorio de Enfermedades Osteoarticulares e Inmunológicas, Sección de Estudios de Posgrado e Investigación, Escuela Nacional de Medicina y Homeopatía, Instituto Politécnico Nacional, Mexico City 07320, Mexico; fergocha@gmail.com

**Keywords:** *Helicobacter pylori*, glucose-6-phosphate dehydrogenase, kinetic, biochemical characterization

## Abstract

*Helicobacter pylori* (*H. pylori*) has been proposed as the foremost risk factor for the development of gastric cancer. We found that *H. pylori* express the enzyme glucose-6-phosphate dehydrogenase (HpG6PD), which participates in glucose metabolism via the pentose phosphate pathway. Thus, we hypothesized that if the biochemical and physicochemical characteristics of HpG6PD contrast with the host G6PD (human G6PD, HsG6PD), HpG6PD becomes a potential target for novel drugs against *H. pylori*. In this work, we characterized the biochemical properties of the HpG6PD from the *H.* *pylori* strain 29CaP and expressed the active recombinant protein, to analyze its steady-state kinetics, thermostability, and biophysical aspects. In addition, we analyzed the HpG6PD in silico structural properties to compare them with those of the HsG6PD. The optimal pH for enzyme activity was 7.5, with a T_1/2_ of 46.6 °C, at an optimum stability temperature of 37 °C. The apparent *K_m_* values calculated for G6P and NADP^+^ were 75.0 and 12.8 µM, respectively. G6P does not protect HpG6PD from trypsin digestion, but NADP^+^ does protect the enzyme from trypsin and guanidine hydrochloride (Gdn-HCl). The biochemical characterization of HpG6PD contributes to knowledge regarding *H. pylori* metabolism and opens up the possibility of using this enzyme as a potential target for specific and efficient treatment against this pathogen; structural alignment indicates that the three-dimensional (3D) homodimer model of the G6PD protein from *H. pylori* is different from the 3D G6PD of *Homo sapiens*.

## 1. Introduction

*Helicobacter pylori* (*H. pylori*) is a Gram-negative bacterium that colonizes the human upper gastrointestinal tract. In general, people that acquire the infection could experience the development of gastritis, the most severe clinical manifestations of which are the generation of peptic ulcers, distal gastric adenocarcinoma, and mucosa-associated lymphoid tissue lymphoma [1,2,3,4,5,6,7,8]. It is estimated that *H. pylori* has infected over 50% of the world’s population; moreover, the infection rate has reached 10–80% in the pediatric population [9]. However, a precise estimation of the age of infection by *H. pylori* is difficult to establish in young children [10]. The International Agency for Research on Cancer declared that *H. pylori* is the principal etiological worldwide agent of gastric cancer and is a type-I carcinogen [11].

There are no exclusive drugs to eradicate *H. pylori,* but the multidrug gold-standard treatment achieves eradication rates of over 90%. In children and adults, triple therapy is recommended, combining two or three antibiotics (clarithromycin, amoxicillin, and metronidazole) and a proton pump inhibitor, such as omeprazole or bismuth salts [12,13]. However, there are increasing and alarming reports of antibiotic-resistant strains, leading to ineffective therapy; for example, resistance to clarithromycin or metronidazole results in *H. pylori* strains that are challenging to eradicate [14]. These findings imply that new therapeutic strategies are urgently needed to treat this infection.

A current model in drug development is rational drug design, focusing on the pathogen’s metabolism. In this context, a rational drug design will identify one or a set of therapeutic targets that are essential to a pathogen’s metabolism, making them targets for evaluating the success of specific drugs [15,16]. Therefore, the structural and functional characterization of target proteins is an essential step in drug development, allowing the subsequent characterization of the potential effect for generating more selective, effective, and efficient new drugs [17]. In this respect, an enzyme of particular interest is the glucose-6-phosphate dehydrogenase (G6PD) in the metabolism of *H. pylori*. *H. pylori* is capable of metabolizing various different carbon sources, especially amino acids and glucose [18,19], efficiently using them via the Entner-Doudoroff pathway (EDP) or the pentose phosphate pathway (PPP) [18,19,20,21,22] to synthesize triose phosphates and ribose phosphates with the generation of reducing power [23,24]. Although *H. pylori* can achieve the glycolytic breakdown of glucose, it has been reported that it lacks the glycolytic enzymes phosphofructokinase (PKF) and pyruvate kinase, affecting its ability to extract energy from hexoses. EDP compensates for the absence of PFK, allowing glucose carbons to be shunted from PPP to EDP, producing glyceraldehyde 3-phosphate and pyruvate [19]. In addition, the enzymes of PPP are present in *H. pylori*, except for 6-phosphogluconate dehydrogenase (6PGDH), which makes the oxidative branch of the pathway incomplete [21]. After that, glucose is metabolized by PPP, which involves the participation of the enzyme glucose-6-phosphate dehydrogenase (G6PD), catalyzing the first rate-limiting activity in the oxidative branch, showing its potential as a target for rational drug design.

Therefore, in this work, we report the cloning and heterologous expression of an active recombinant G6PD from the *Helicobacter pylori* strain 29CaP, isolated from a Mexican patient with gastric cancer [25]. This study allowed us to purify the predicted protein and analyze the detailed steady-state kinetics, thermostability, and biophysical characteristics of the HpG6PD enzyme. Finally, we use computer modeling techniques to describe the structural features that clearly distinguish HpG6PD from the human G6PD enzyme (HsG6PD), proposing this *H. pylori* enzyme as a potential therapeutic target.

## 2. Materials and Methods

### 2.1. Strain and Plasmids

*E. coli* BL21(DE3)Δzwf:kan^r^ was used for all cloning and expression procedures. The Puc57 vector containing the *zwf* gene was purchased from GeneScript (GenBank: ALM79592.1). The pET3aHisTEVP vector was previously obtained for an earlier study [26], and the pET3aHisTEVP-*zwf* constructed in this project was sequenced in the Instituto de Biotecnología, UNAM, Cuernavaca City, Morelos, Mexico.

### 2.2. Reagents

The restriction enzymes *Nde*I, *Bam*HI, and T4 DNA ligase were purchased from Thermo Scientific (Mannheim, Germany), and the Promega Corporation (Madison, WI, USA). The GeneJET Gel Extraction Kit was obtained from Thermo Scientific (Hudson, NH, USA). Profinity IMAC Resins and Gel Filtration Standard, used as a lyophilized mixture of molecular-weight markers, ranging from 1350 to 670,000 Da, were purchased from Bio-Rad (Hercules, CA, USA). Isopropyl β-d-1-thiogalactopyranoside (IPTG), Tris-HCl, PMSF, β-mercaptoethanol, glycerol, NaCl, MgCl_2_, imidazole, dithiothreitol (DTT), Coomassie brilliant blue R, G6P, NADP^+^ substrates, ampicillin, and kanamycin antibiotics were obtained from Sigma-Aldrich (St. Louis, MO, USA). The Midori Green Advance dye was purchased from NIPPON Genetics Europe, Dueren, Germany.

### 2.3. Alignment of Sequences

The glucose-6-phosphate dehydrogenase sequence from *H. pylori* and other bacterial and eukaryotic G6PD sequences were obtained from the NCBI database. Multiple sequence alignment was performed using Unipro UGENE v. 42.0 software [27], and the GeneDoc program was used to visualize the alignment. Here, we indicate the position of each residue in the sequence of each G6PD enzyme. We also indicate the positions of the same residue (in parentheses) according to the HpG6PD sequence.

### 2.4. Construction of pET3aHisTEVP-zwf Vector

The Puc57 vector carrying the *H. pylori zwf* gene was digested using the *Nde*I and *Bam*HI enzymes. The resulting digestion was analyzed in 1.0% agarose gel via electrophoresis and was subsequently stained with Midori Green Advance, then, finally, analyzed using the MultiDoc-It (UVP) equipment. The fragment (*zwf* gene) comprising 1278 base pairs (bp) was purified using a GeneJET Gel Extraction Kit, following the manufacturer’s protocol. Afterward, the gel fragment was purified and ligated into the expression vector pET3aHisTEVP, to generate pET3aHisTEVP-*zwf* (Figure S1). The resulting vector was sequenced in the Unidad de Síntesis y Secuenciación from the Instituto de Biotecnología, UNAM, Cuernavaca City Morelos. The results were compared with the BLAST sequencing database (NCBI), resulting in 99 hits with 100% identity and an E-value of 0.0 for *H. pylori*. The pET3aHisTEVP-*zwf* plasmid containing the *zwf* gene insert was used to transform *E. coli* BL21(DE3)Δzwf:kan^r^.

### 2.5. Expression and Purification of the G6PD Protein from Helicobacter pylori

The BL21(DE3)Δzwf:kan^r^ transformed with pET3aHisTEVP-*zwf* plasmid was cultured in 50 mL of Luria Bertani (LB) medium with selection antibiotics and incubated on a rotary shaker at 37 °C for 24 h. Cell cultivation was then scaled up to 2 L on LB medium with selection antibiotics, incubated on a rotary shaker at 37 °C, and continued until the culture reached an optical density of O.D_600 nm_ 1.0. At that point, an induction was performed, with 0.3 mM IPTG incubated on a rotary shaker at 25 °C for 18 h. Then, the cells were centrifuged at 6000× *g* at 4 °C for 30 min. Next, the cells were disrupted by sonication (ten cycles of 45 s in an ice bath) with lysis buffer (500 mM Tris-HCl, at pH 7.6, 3 mM MgCl_2_, 0.5 mM PMSF, 0,1% β- mercaptoethanol, and 5% glycerol) using the Branson Sonifier 450 from VWR Scientific (Radnor, PA, USA). Finally, the product was centrifugated at 10,000× *g* at 4 °C for 30 min and the soluble fraction, known as the crude extract, was used to purify the HpG6PD protein.

The HpG6PD protein was purified via immobilized metal affinity chromatography (IMAC). The *H. pylori* crude extract was loaded into a Ni Sepharose High-Performance column (GE Healthcare, Chicago, IL, USA), previously equilibrated with 5 column volumes (CV) of equilibrium buffer (50 mM Tris-HCl, at pH 8.0) after it was washed with 10 CV of washing buffer (50 mM Tris-HCl, at pH 8.0, 150 mM NaCl, 50 mM imidazole, and 2 mM DTT). Then, the protein was eluted with an equilibrium buffer plus 250 mM of imidazole [28]. The imidazole was removed from the sample by five consecutive dilutions with equilibrium buffer and it was then concentrated using Amicon YM-15 filtration tubes (Millipore, Burlington, MA, USA). The (His)6-tag sequence was removed from the HpG6PD protein using recombinant tobacco virus protease (TEVP), according to a previously reported protocol [29]. Protein purity was verified by 10% sodium dodecyl sulfate-polyacrylamide gel electrophoresis (SDS-PAGE) [30] and Colloidal Brilliant Coomassie Blue R-250 was used to stain the gel. The protein concentration was determined by the Lowry method with Folin-phenol reagent [31], using bovine serum albumin as a standard.

### 2.6. Enzymatic Activity Assay

The activity of HpG6PD was determined spectrophotometrically at 340 nm following NADPH formation using a Cary50 UV-Vis spectrophotometer (Varian, Crawley, UK). Standard activity assays were performed with 1 mL of the standard reaction mixture (Tris-HCl, pH 7.6, 3 mM MgCl_2_, 1 mM glucose 6-phosphate, and 150 μM NADP^+^), then 1 µg of purified HpG6PD was added to start the reaction.

### 2.7. Oligomeric Composition Analysis by Molecular Exclusion 

The oligomeric state of the native HpG6PD protein was determined by gel filtration chromatography. The HpG6PD protein was adjusted at 0.4 mg/mL in 400 µL of phosphates buffer, pH 7.35, and applied to the Sephacryl^TM^ S-200 HR HiPrep^TM^ 16/60 column coupled to the ÄKTA pure FPLC system (GE Healthcare, Chicago, IL, USA) and eluted with equilibrium buffer (50 mM phosphate buffer pH 7.35) at a flow rate of 0.5 mL/min. The column was calibrated with gel filtration standard with molecular masses ranging from 1350 to 670,000 daltons.

### 2.8. Determination of the Functional Parameters of the G6PD Protein of Helicobacter pylori

#### 2.8.1. Enzyme Stability, pH, and Optimal Temperature

The effect of pH on the activity of purified HpG6PD was assessed within a pH range of 3.0 to 10.0 in either 50 mM McIlvaine buffer (pH 3.0–6.0), MES buffer (pH 6.0–6.75), HEPES buffer (pH 6.75–8.0), Tris buffer (pH 8.0–9.0), or glycine buffer (pH 9.0–10) under enzymatic activity assay conditions, using 1 mM G6P and 150 mM NADP^+^. First, the activity was measured with 1 mg of HpG6PD protein. Then, the optimal pH was determined by obtaining the residual activity, with the highest value taken as 100. In addition, the pH stability of the purified HpG6PD was assessed by incubating the enzyme in the four different buffer systems mentioned above at 25 °C for 24 h. Then, the residual G6PD activities were determined as previously described [28].

The effect of temperature on HpG6PD activity was determined by measuring the G6PD activity at different temperatures, ranging from 4 °C to 70 °C, and an optimum pH using G6P and NADP^+^ substrates. Initial velocities were expressed as a percentage, and the highest activity was set at 100%. Finally, the stability at the temperature of HpG6PD was assessed at different temperatures. The residual activity of the enzyme was measured after incubating it for 20 min at different temperatures, ranging from 37 to 65 °C. The protein was adjusted at a final concentration of 0.2 mg/mL in Tris-HCl buffer (100 mM Tris-HCl, 2 mM EDTA, pH 7.4), then the samples were cooled on ice for 4 min and the residual activity enzyme was measured under standard enzyme assay conditions, along with the addition of 1 µg of the incubated protein.

#### 2.8.2. Determination of Kinetic Parameters

The experimental steady-state kinetic parameters of the HpG6PD were determined for the G6P and NADP^+^ substrates. The initial velocity data for the NADP^+^ substrate were obtained by varying the NADP^+^ concentration (2.5 to 200 μM) and maintaining G6P at a saturating concentration (500 μM). In comparison, the initial velocity data for the G6P substrate were obtained by maintaining NADP^+^ at a saturating concentration (200 μM) and a G6P concentration in the range of 2.5 to 300 μM. The reaction was initiated by adding 1 μg of the purified protein. The Michaelis–Menten kinetic constants were determined via nonlinear regression calculations, as reported in a previous study [32]. An international unit (IU) of G6PD activity was defined as the amount of enzyme required to produce 1 µmol of NADPH per minute, per mg of protein.

### 2.9. Spectroscopic Characterization

#### 2.9.1. Far-UV Circular Dichroism

The secondary structure of HpG6PD was analyzed by circular dichroism (CD) using a Jasco J-810^®^ spectropolarimeter (163–900 nm) equipped with a Peltier thermostated cell holder for temperature control and a constant flow of nitrogen. The far-UV CD spectra were recorded at far-UV from 200 to 260 nm and at 1 nm intervals, using the 0.1 cm path length of a rectangular quartz cuvette. The assays were performed with a protein concentration of 0.5 mg/mL in 50 mM phosphate buffer and were performed in duplicate at 25 °C.

#### 2.9.2. Thermal Stability, Monitored by Circular Dichroism 

The effect of temperature on the global stability of the HpG6PD protein was evaluated via a thermal stability assay by circular dichroism. The protein was adjusted to 0.4 mg/mL with P buffer (0.05 M potassium phosphate buffer pH 7.4) and evaluated by increasing the sample temperature from 35 to 85 °C at constant heating rates of 1 °C/2.5 min. The changes in the molar ellipticity (Φ) at 222 nm were monitored with a Jasco J-810^®^ spectropolarimeter. The data that were obtained fitted Bolzman’s sigmoid equation with the Origin^®^ program. In addition, the thermal denaturation transition of the protein was evaluated to obtain the *T*_m_ value (the temperature at which the HpG6PD is 50% in the denatured state and 50% in the native state). The assay was performed in triplicate.

### 2.10. Evaluation of the Stability of the Recombinant HpG6PD Protein

#### 2.10.1. Stability Analysis of HpG6PD Enzyme Activity

To determine if the physiological substrates (G6P and NADP^+^) affect the stability of the HpG6PD enzyme, we performed thermal inactivation and susceptibility to protease assays and ascertained the effect of guanidine hydrochloride. For thermal stability, the HpG6PD protein was adjusted at 0.2 mg/mL in Tris-HCl buffer (100 mM Tris-HCl, 2 mM EDTA, pH 7.4), then incubated with three different concentrations of G6P and NADP^+^ (10, 100, and 500 μM) for 2 h at 37 °C. The susceptibility of HpG6PD to protease was performed by adjusting the HpG6PD protein concentration to 0.2 mg/mL and incubating it with different trypsin concentrations, ranging from 0 to 1 mg/mL in three different concentrations of physiological substrates (10, 100, and 500 μM), then incubating for 2 h at 37 °C. Before the residual activity was measured, the reaction was stopped with 5 mM of PMSF, as previously reported by Cortes-Morales et al. [33]. Finally, the stability of HpG6PD was evaluated, with a concentration of 0.2 mg/mL of protein and by varying the concentration of Gdn-HCl, ranging from 0 to 1 M; the protein was then incubated at 37 °C for 2 h. The value of C_1/2_ (the concentration of Gdn-HCl necessary for the protein to lose half of its activity) was determined, as previously reported by Ramirez-Nava et al. [34]. In addition, to evaluate whether the physiological substrates G6P and NADP^+^ exert a protective effect on HpG6PD in the presence of Gdn-HCl, the HpG6PD was incubated with three different concentrations of G6P or NADP^+^ (10, 100, and 500 μM). In all assays, the residual activity was measured, and the assays were performed in triplicate.

#### 2.10.2. Fluorescence Spectroscopy

Fluorescence spectroscopy of HpG6PD was conducted using a Perkin-Elmer LS-55 Spectrofluorometer (Perkin–Elmer, Wellesley, MA, USA) and a quartz cell with a path length of 1.0 cm at 25 °C. The excitation wavelength was 295 nm, with excitation and emission slits of 4.5 and 3.7 nm, respectively. The protein was adjusted to a 0.1 mg/mL concentration in phosphate buffer. As a result, the fluorescence spectrum of the protein was obtained in a wavelength range of 310–500 nm. In addition, intrinsic tryptophan fluorescence was monitored at different Gdh-HCl concentrations (from 0 to 2 M) and incubated at 37 °C for 2 h. In both trials, the data of the final spectra were corrected by subtracting the blank, which was obtained by measuring the intrinsic fluorescence intensity of the phosphate buffer. The assay was performed in triplicate.

## 3. Results and Discussion

### 3.1. Alignment of G6PDs Proteins

The HpG6PD amino acid sequence was compared with other G6PD proteins. We included ten sequences from the NCBI database to perform the alignment in this work. HpG6PD shares 43%, 38%, 36%, 34%, and 27% of identity with *Campylobacter jejuni*, *Neisseria gonorrhoeae*, *Pseudomonas aeruginosa*, *Trypanosoma* *cruzy*, and *Homo sapiens* G6PD, respectively. The HpG6PD amino acid sequence was compared with the G6PD *Homo sapiens* sequence; we found 27% of identity via a BLAST protein analysis against the Protein Data Bank (PDB) records. In addition, to analyze the structural similarity between the enzymes HpG6PD and HsG6PD, and to establish the structural equivalences according to their shape and three-dimensional conformation, the representative tridimensional model of HpG6PD was aligned with the HsG6PD structure (PDB entry 2BH9), using Chimera 1.14.2. A root mean square deviation (RMSD) value of 1.423 Å was obtained for 406 Cα atoms and the Q-score value was 0.648. The RMSD value is a commonly used measure of similarity between two protein structures; the smaller the RMSD, the more similar the two structures. In the case of the Q score, a zero value represents completely dissimilar or un-superimposed structures and 1 represents identical structures. Therefore, the results obtained from the structural alignment indicate that the three-dimensional (3D) homodimer model of the G6PD protein from *H. pylori* [35] is different from the 3D G6PD of *Homo sapiens*, making the HpG6PD protein a potential pharmacological target (Figure S2).

Multiple-sequence alignment of the G6PDs proteins revealed three conserved fragments: a nine-residue peptide (RIDHYLGKE, residues 162–170 of the HpG6PD enzyme), a nucleotide-binding fingerprint (GxGGDLA, residues 10–16 of the HpG6PD enzyme), and the sequence EKP*x*G (residues 134–138 of the HpG6PD enzyme).

**Figure 1:**
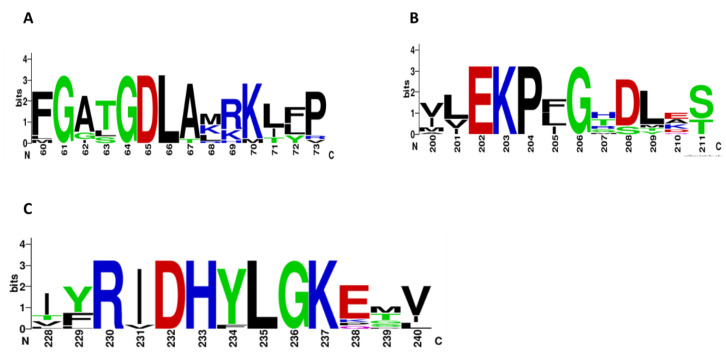
Multiple-sequence alignment of the G6PDs’ amino acid sequences. Residues that have been reported to interact with the substrates (**A**) NADP^+^ and (**B**,**C**) G6P are shown. The sequence alignment was carried out using the Uniprot UGENE software [27]. The comparison of conserved regions observed in the amino acid sequence alignment of G6PDs was conducted using the WebLogo3 online program.

As seen in Figure S3, the HpG6PD amino acid sequence contains the NADP^+^ fingerprint, 10-GxGGDLA-16 (the amino acid number corresponding to the G6PD sequence from HpG6PD, shown in the green background); this amino acid sequence is a highly conserved motif in G6PD proteins [36,37]. In addition, the HpG6PD protein has the pentapeptide-conserved fragment that is described as motif 134-EKP*x*G-138 (the amino acid number corresponding to the HpG6PD sequence), which is well conserved among all G6PDs (Figure 1), wherein the center is the Pro136; this residue is directly involved in the correct positioning of the substrate and coenzyme pockets [38]. Finally, the third fragment in the nine-residue peptide 162-RIDHYLGKE-170, Asp164, His165, and Lys169 are important in G6P binding and catalysis [39], while Lys205 has been implicated in binding and catalysis in the human enzyme [40]. Therefore, the presence of the three conserved fragments suggests that the HpG6PD is potentially functional.

### 3.2. Overexpression and Purification of Recombinant HpG6PD 

**Figure 2:**
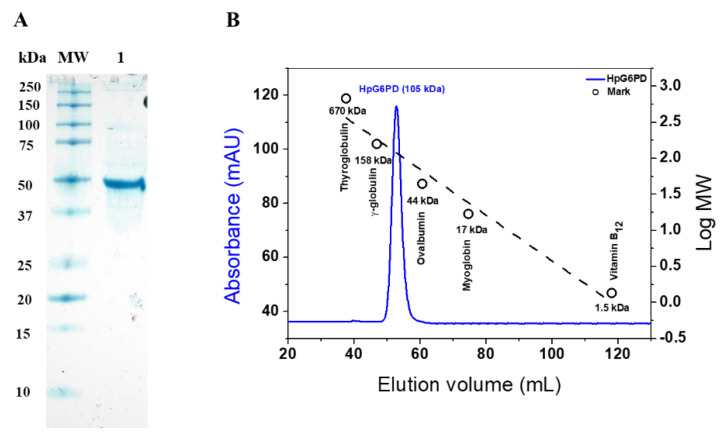
Purification and molecular exclusion chromatogram of the recombinant HpG6PD enzyme. (**A**) SDS-PAGE analysis of the recombinantly produced HpG6PD. The protein was analyzed on 12% polyacrylamide gel electrophoresis (SDS-PAGE). M: Bio-Rad kaleidoscope MW marker. Lane 1: 20 micrograms of purified HpG6PD protein. The gels are representative of three independent experiments. (**B**) FPLC chromatogram of native HpG6PD and standard proteins (black dashed line, Bio-Rad), as follows: bovine thyroglobulin, 670 kDa; bovine γ-globulin, 158 kDa; ovalbumin, 44 kDa; horse myoglobin, 17 kDa; and vitamin B12, 1.3 kDa.

The recombinant HpG6PD protein was overexpressed when using the *E. coli* strain BL21(DE3)Δzwf:kan^r^; under the conditions used, the recombinant protein was obtained in the soluble fraction and was purified by immobilized metal affinity chromatography (IMAC), with yields of 40% and a total protein of 9.8 mg. To determine the biochemical and functional parameters of the HpG6PD protein, the His-tag was removed by specific TEVP protease activity, as previously described [41]. The purity of the enzyme was analyzed by electrophoresis, using 12% polyacrylamide gel under denaturing conditions (SDS-PAGE). As seen in Figure 2A, a single band with a relative molecular weight (MW) of 50 kDa and high purity was obtained. According to the GenBank accession, this relative MW agrees with the theoretical MW of 49 kDa obtained by the Expasy ProtParam tool (https://web.expasy.org/protparam/ (accessed on 24 February 2022), according to the GenBank accession number ALM79592.1. Furthermore, this result is in agreement with previous reports of G6PD proteins from other prokaryotic organisms such as *Gluconacetobacter diazotrophicus*, *Leuconostoc mesenteroides*, *Thermotoga maritima*, and *Pseudomonas aeruginosa* [34,42,43,44], where the purified G6PDs proteins presented MWs of around 50 kDa, unlike G6PD proteins from eukaryotic organisms, which show relative MWs in the range of 60 to 75 kDa. Subsequently, with the purified HpG6PD enzyme, we performed the biochemical assays described below.

### 3.3. Determination of the Oligomeric State of the Recombinant HpG6PD

**Figure 3:**
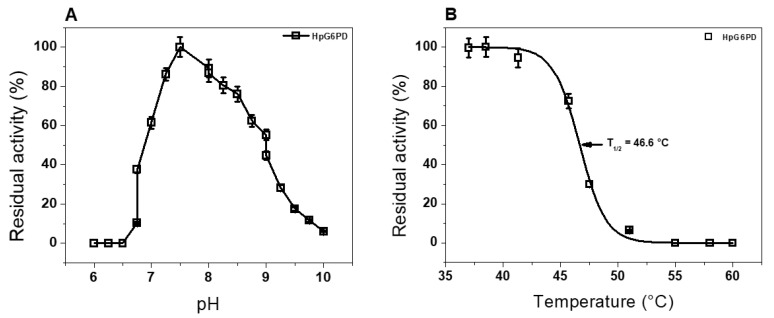
The effect of pH and temperature on the catalytic activity of the HpG6PD protein. (**A**) The effect of pH on the activity of the HpG6PD protein. The enzyme was incubated in four system buffers with a pH ranging from 6.0 to 10.0. The non-enzymatic reduction of NADP^+^ was also measured at each pH value, to subtract the value obtained at each experimental point. (**B**) The effect of temperature on the activity of HpG6PD protein. In both experiments, the protein was adjusted to 0.2 mg/mL, and 1 µg of total protein was used to measure the residual activity. Data are presented as mean values ± Standard Error Media (SEM) of 3 independent experiments.

The gel chromatography technique was used to determine the oligomeric state of HpG6PD in solution. The recombinant enzyme was eluted in a single main peak with a retention volume of 52 mL (Figure 2B), with catalytic activity for G6PD. In addition, the formation of oligomeric aggregates of the recombinant protein in solution was not observed. The Bio-Rad’s Gel Filtration Standard was loaded under the same conditions as the HpG6PD; the chromatogram revealed that the HpG6PD eluted in the homodimer form, as demonstrated in Figure 3B, with a single peak that corresponded to a size of 105 kDa, which is close to the MW expected from the amino acid sequence (49,000 × 2 = 98 kDa). This result is consistent with those reported for other G6PDs from prokaryotic organisms, such as *Leoconostoc mesenteroides* and *Haloferax volcanii*, which were reported as homodimers in solution [45,46]. Despite other G6PDs that have been reported as homotetramers in solution, such as *Gluconoactobcter diazotrophicus* and *Pseudomonas aeruginosa* [34,44], the G6PD of *Homo Sapiens* has been reported as a dimer and a tetramer, and the dimer has greater catalytic activity than the tetramer [32].

### 3.4. Determination of the Functional Parameters of the HpG6PD Protein

#### 3.4.1. Effect of pH and Temperature Stability

To determine the effect of pH on the purified recombinant HpG6PD protein activity, we carried out an activity analysis at various pH values, ranging from 3.0 to 10.0. The curve obtained in this assay (Figure 3A) showed a classical bell-shaped curve, as previously reported for different G6PDs enzymes, in which we observed the highest point of activity (100%) at a pH of 7.5 [34,44,47,48,49]. Interestingly, the enzyme’s activity decreases until it loses activity (0%) at a pH of 10.0. Based on these results, we found that the optimal pH value for activity of the recombinant HpG6PD enzyme was 7.5. This optimum pH value is like that previously reported for other G6PDs from prokaryotic organisms such as *Gluconoacetobacter diazotrophicus*, *Pseudomonas aeruginosa*, and *Escherichia coli*; these showed optimal pH values of 7.5 to 8.0 [34,44,50]. In light of these results, the following functional studies were performed at pH 7.5.

In addition, the residual catalytic activity of the enzyme after incubation at different temperatures was evaluated, to determine the temperature stability of the HpG6PD enzyme. As Figure 3B shows, the enzyme shows a percentage of residual activity between 100% and 95% in a temperature range from 37 °C to 41 °C. However, when the enzyme is exposed to a temperature of 45 °C, it shows only a residual activity of 75%; when the temperature is increased to 47.5 °C, the enzyme has a residual activity of 30%. Finally, the enzyme loses its catalytic activity when exposed to a temperature of 60 °C (Figure 3B). The temperature stability plot showed that the temperature at which the enzyme loses 50% of its original activity (T_1/2_) was 46.6 °C. Furthermore, we found that temperature stability in terms of HpG6PD activity is similar to that previously reported for G6PD that was purified from the Gram-negative bacterium *Sphingomonas* sp., in which the enzyme retains 100% of its activity in a temperature range from 30 °C to 40 °C [51]. However, the HpG6PD enzyme showed lower temperature stability than the G6PD of the bacteria *G. diazotrophicus*, in which its G6PD enzyme retains 100% of its catalytic activity in a broader temperature range from 37 °C to 50 °C [34]. These results allowed us to report an optimal stability temperature for HpG6PD of 37 °C; therefore, the functional assays were carried out at this temperature.

#### 3.4.2. Determination of Kinetic Parameters

**Figure 4:**
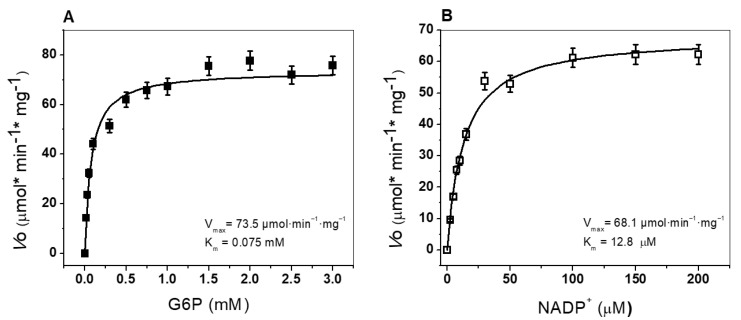
Saturation curves for the recombinant HpG6PD enzyme for the substrates (**A**) G6P and (**B**) NADP^+^. The initial velocity data for each point were obtained by varying the concentration of the substrates with a fixed and saturating concentration of NADP^+^ or G6P, depending on the assay. These data were fitted to the Michaelis–Menten equation, using nonlinear regression calculations.

The enzymatic activity of the recombinant HpG6PD enzyme was assessed following the standard protocol, by detecting the conversion of the substrate NADP^+^ into NADPH. Thus, the steady-state kinetic parameters of the recombinant HpG6PD protein were obtained for both substrates (G6P or NADP^+^). As seen in Figure 4, the HpG6PD enzymatic activity showed a hyperbolic function for the two physiological substrates. The apparent *K_m_* values calculated for G6P and NADP^+^ were 75.0 and 12.8 µM, respectively (Table 1). Furthermore, the kinetic parameters were compared with other G6PDs from prokaryotic and eukaryotic organisms. We found that the HpG6PD *K_m_* for the G6P substrate is similar to that obtained in the G6PD of *G. diazotrophicus* [34], *Trypanosoma cruzy* [52], and camel liver [47]. In the case of the substrate NADP^+^, a *K_m_* value of 12 µM was calculated for HpG6PD; this value is similar to that reported for the G6PD of *G. lamblia* [53], *T. cruzy* [52], and *B. malayi* [49], where the *K_m_* values for NADP^+^ ranged from 14 to 16 μM (Table 1). Finally, the catalytic constant (*k_cat_*) was determined, with a value of 70 s^−1^. As can be seen in Table 1, the recombinant HpG6PD protein has a lower *k_cat_* (70 s^−1^) value than the G6PD enzymes from *G. diazotrophicus* (293181 s^−1^), *P. aeruginosa* (540 s^−1^), *A. orizae* (1000 s^−1^), *T. maritima* (35000 s^−1^), and *Homo sapiens* (233 s^−1^) [34,43,44]. With all these enzymatic kinetic parameters, in the future, we could determine the mechanism of action (inhibition type) of the synthetic compounds in terms of HpG6PD activity, identifying it as a potential therapeutic target.

### 3.5. Spectroscopic Characterization

#### 3.5.1. Circular Dichroism (CD) Assay

**Figure 5:**
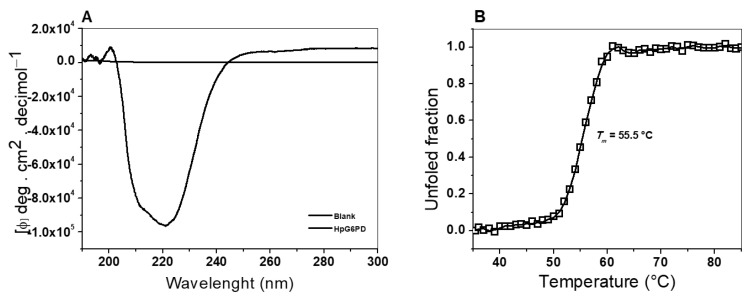
Circular dichroism assay (CD) and thermal stability assessment of the HpG6PD enzyme. (**A**) The far-UV circular dichroism spectrum of HpG6PD in its native state. (**B**) Analysis of thermal stability via CD. CD monitored the changes in the signal at 222 nm, as the temperature increased from 35 °C to 85 °C. In both experiments, the HpG6PD protein was incubated at 0.5 mg/mL in 25 mM phosphate buffer (pH 7.4). Both experiments are representative of three independent experiments.

The secondary structure of the recombinant HpG6PD was analyzed by far-UV circular dichroism (CD) spectroscopy. This allowed us to determine the composition of the α-helices and β-sheets present in the native state of the protein. As seen in Figure 5A, the CD spectrum of HpG6PD showed minimal absorption peaks at 208 and 222 nm, indicating that the protein has α-helices and β-sheets in its structure. In addition, an important finding was that the CD spectrum showed a higher absorption at 222 nm than the absorption at 208 nm, indicating that the HpG6PD protein in the native state contains a higher content of α helices to β sheets. Additionally, this spectrum resembles the spectra reported for other G6PD enzymes in prokaryotic and eukaryotic organisms. This observation implies that the secondary structure content is comparable to that of other G6PDs [29,32,58]; these other studies have reported that the N-terminus (amino acids 1–164) contained the β-α-β Rossmann-type folding domain, which contains the binding sites of NADP^+^ and β-D-glucose-6-phosphate (G6P).

#### 3.5.2. Thermal Stability Assay, Followed by CD

A thermal stability assay was performed to analyze the structural stability of the HpG6PD protein by circular dichroism (CD), following changes to the α–helices at 222 nm. In Figure 5B, we show the denaturation profiles of HpG6PD in phosphate buffer at pH 7.4. The profile obtained under these experimental conditions showed the two states of thermal denaturation and was calculated at a *T*_m_ value of 55.5 °C. Therefore, considering these *T*_m_ values, we found that HpG6PD is a more stable protein than the G6PD of the Gram-negative bacteria, *G. diazotrophicus* (*T*_m_ = 50.6 °C) [34]. However, HpG6PD is more sensitive than the recombinant human WT HsG6PD, in which a *T*_m_ of 59.5° was reported [32]. Finally, the *T*_m_ value of HpG6PD was similar to that observed in the G6PD::6PGL fusion enzymes of parasites such as *G. lamblia* and *T. vaginalis* (*T*_m_ = 57 °C and 54 °C), respectively [40,52].

### 3.6. Evaluation of the Stability of the Recombinant HpG6PD Protein

#### 3.6.1. Thermal Inactivation Assay

**Figure 6:**
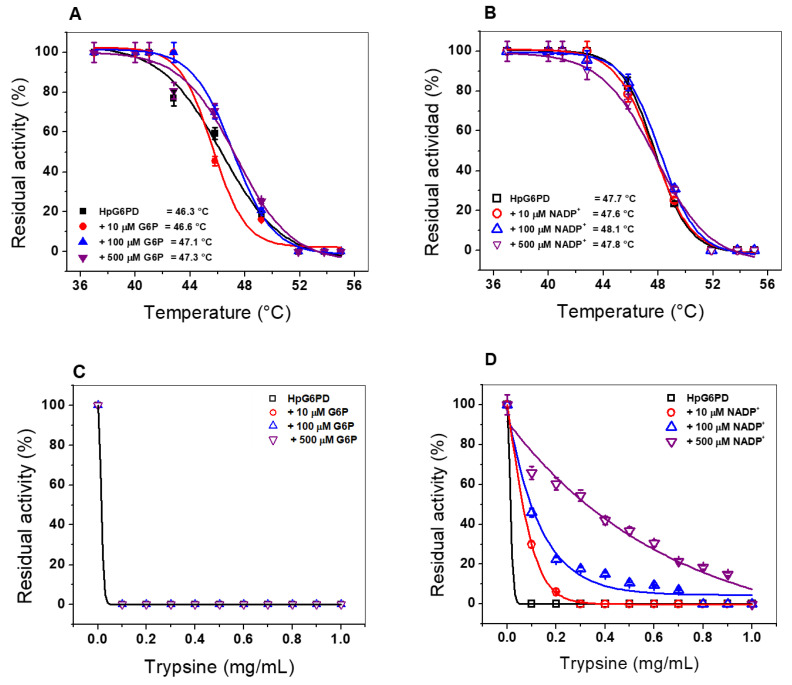
Evaluation of the stability of the HpG6PD protein, via a thermal inactivation assay in the absence and presence of different concentrations of G6P (**A**) and NADP^+^ (**B**). (**C**) Digestion of the recombinant HpG6PD protein with different concentrations of trypsin (0 to 1 mg/mL), in the presence of three concentrations of G6P (10, 100, and 500 μM) and (**D**) NADP^+^ (10, 100, and 500 μM). In all the assays, the protein was adjusted to a concentration of 0.2 mg/mL in phosphate buffer, pH 7.35, and was incubated for 2 h at 37 °C, after which the residual activity was measured. The assay was initiated with the addition of 1 µg of total protein. Data are presented as mean values ± SEM of 3 independent experiments.

To evaluate the stability of the active site of the HpG6PD, we performed a thermal inactivation assay. Thus, we evaluated the enzymatic active-site stability of HpG6PD, along with the effect of three concentrations of the substrates G6P and NADP^+^ on the thermal stability of the enzyme. Figure 6A,B shows the HpG6PD temperature stability versus residual activity graphs, showing the temperature at which the enzyme loses 50% of its activity (*T*_50_) after incubation for 20 min. The average HpG6PD *T*_50_ value in the absence of G6P was 46.3 °C. This value did not change when 10 µM of G6P was added, and when the concentration of G6P was increased to 100 and 500 µM, conditions in which *T*_50_ only increased by 1 °C (*T*_50_ at 500 µM = 47.3 °C) (Figure 6A). In contrast, the *T*_50_ value shown by the enzyme in the absence of NADP_+_ was 47.7 °C; we found that the thermal stability of the enzyme did not improve with any of the NADP^+^ concentrations tested (Figure 6B). These results indicate that the active site is not stabilized by either G6P or NADP^+^, as previously observed for human HsG6PD and in parasitic organisms, such as *Giardia lamblia* and *Trichomonas vaginalis*, where an increase from 6 °C to 10 °C was observed in the presence of 500 µM NADP^+^ [32,41].

#### 3.6.2. Susceptibility of the HpG6PD Enzyme to Trypsin Proteolysis

Another approach to evaluate protein stability is by assessing its susceptibility to trypsin digestion, a method frequently used to test resistance to proteolysis [33]. Figure 6C shows the effect of trypsin on HpG6PD in the presence and absence of the G6P substrate; it is clear that the enzyme loses 100% of its activity from the lowest concentration of trypsin tested (0.1 mg/mL); this susceptibility does not change, despite the addition of increasing concentrations of G6P. This result indicates that the G6P substrate does not have a protective effect on HpG6PD, as reported for the G6PD enzyme from *Pseudomonas aeruginosa* [44].

**Figure 7:**
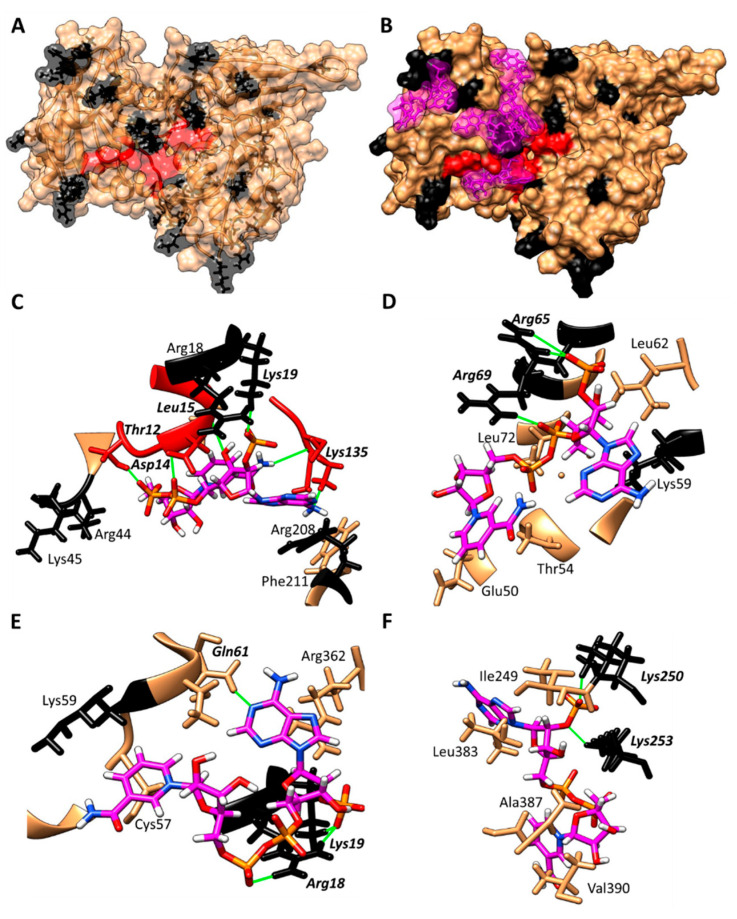
Docking study between HpG6PD and the NADP^+^ molecule. (**A**) Model of the HpG6PD enzyme, showing the Lys and Arg residues in black at the points where trypsin can hydrolyze. (**B**) Results of the docking study, showing four zones of possible interaction of the NADP^+^ molecule with HpG6PD; the NADP^+^ molecule is shown in a magenta color. (**C**–**F**) Zoomed-in view of the predicted interaction zones between the HpG6PD enzyme and the NADP^+^ molecule; hydrogen bridges are shown in green, while the amino acids involved are shown in bold italics.

Regarding the effect of the coenzyme NADP^+^ on HpG6PD protein proteolysis, we found that this substrate exerts a protective effect against proteolysis. As shown in Figure 6D, in the absence of the NADP^+^, HpG6PD loses 100% of its activity when exposed to 0.1 mg/mL trypsin. However, when HpG6PD was incubated in the presence of 10 µM of the substrate NADP^+^, we observed that at 0.1 mg/mL trypsin, the enzyme retained 30% of its activity (Figure 7D). When the concentration of NADP^+^ was increased to 100 µM, the HpG6PD retained 50% of its activity when exposed to 0.1 mg/mL trypsin. Finally, when 500 µM NADP^+^ was added, 0.4 mg/mL trypsin was needed for the enzyme HpG6PD to lose 50% of its activity. These results indicate that the HpG6PD protein resists digestion with trypsin in the presence of the coenzyme NADP^+^, suggesting a protective effect by the NADP^+^ molecule. Notably, the trypsin digestion assay has been widely used to assess the stability of mutants compared to the wild-type enzyme for the recombinant human G6PD enzyme. In this assay, it has been observed that in the presence of the NADP^+^ molecule, human G6PD variants exhibit increased resistance to trypsin digestion; however, in prokaryotic organisms, this effect has not been observed [29,33,58].

To confirm the protective effect of the coenzyme NADP^+^ over the HpG6PD against proteolysis digestion by trypsin, we determined the presence of trypsin cleavage sites in the native protein and performed a blind docking, to establish whether the coenzyme NADP^+^ is binding at the trypsin cleavage sites on HpG6PD. Based on the above tests, we found that the HpG6PD primary sequence contains 40 trypsin cleavage sites, which explains its susceptibility to trypsin hydrolysis (in Figure 7A, the cleavage sites for trypsin are shown in black). Subsequently, a docking study was performed between the HpG6PD model and the NADP^+^ molecule. The results predicted that NADP^+^ has a high affinity for the enzyme’s catalytic site (Figure 7B); we found that the most stable conformer forms up to 7 hydrogen bonds and has a theoretical ΔG of −12.12 Kcal, and that 25% of the conformers are positioned at this binding site. However, four more zones of possible interaction of the NADP^+^ molecule with HpG6PD were found near other trypsin cleavage sites (Figure 7C–F, hydrogen bonds are shown in green); it can be seen that NADP^+^ is positioned near several trypsin cleavage sites. These results indicate that the protective effect of trypsin digestion could probably be due to steric hindrance by the binding of the NADP^+^ molecule to cleavage sites for trypsin in the native HpG6PD protein.

#### 3.6.3. Stability of the HpG6PD Protein in the Presence of Guanidine Hydrochloride (Gdn-HCl)

**Figure 8:**
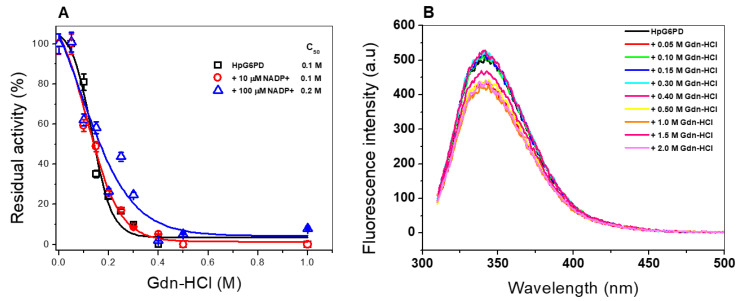
Stability and structural analysis of HpG6PD via intrinsic fluorescence. (**A**) Stability analysis in the presence or absence of Gdn-HCl. (**B**) Intrinsic fluorescence intensity of HpG6PD at different Gdn-HCl concentrations. Assays were performed in triplicate, with standard errors of less than 5%.

Finally, an inactivation assay was performed in the presence and absence of guanidine hydrochloride (Gdn-HCl) to evaluate the stability of the recombinant HpG6PD enzyme. Gdn-HCl is a chaotropic and denaturing agent used to determine the conformational stability of proteins, as it alters the tertiary structure of the protein; consequently, the catalytic activity of the protein is perturbed [58]. As shown in Figure 8A, in the absence of NADP^+^, the specific activity of the recombinant HpG6PD enzyme decreased as the concentration of Gdn-HCl increased, determining a C_1/2_-Gdn-HCl of 0.1 M. Subsequently, when the protein was incubated in the presence of 10 µM NADP^+^, the same behavior was observed. However, a slight protective effect was found with 100 µM NADP^+^, when a value of C_1/2_-Gdn-HCl of 0.2 M was determined. We found that the HpG6PD enzyme is more sensitive to Gdn-HCl, due to the fact that a concentration of 0.1 M of Gdn-HCl decreases its activity to 50%, while in the case of the G6PD enzyme from the Gram-negative bacterium *G. diazotrophicus* and the human G6PD enzyme, a concentration of 0.6 M and 0.45 M, respectively, is needed [33,34].

Although, in the thermal stability assays, the NADP^+^ molecule was not found to exert a protective effect on HpG6PD, in terms of the stability of the G6PD protein in the presence of trypsin and Gdn-HCl, a slight protective effect of the NADP^+^ molecule was observed. As the docking results showed, this protective effect can probably be attributed to the fact that the NADP^+^ molecule binds to the protein in some cavities of the native structure and thereby blocks the interaction of trypsin or Gdn-HCl, preventing these molecules from exerting their proteolytic or denaturation effects on the native structure of the recombinant HpG6PD protein.

#### 3.6.4. Structural Analysis via Intrinsic Fluorescence

An intrinsic fluorescence assay evaluated the effect of Gdn-HCl on the structural stability of the HpG6PD enzyme. As seen in Figure 8B, a decrease in the fluorescence intensity of the protein was detected, caused by the different concentrations of Gdn-HCl tested. HpG6PD shows a fluorescence intensity of 500 arbitrary units (a.u.) in the absence of Gdn-HCl. This decrease in fluorescence is due to the exposure to an aqueous medium of the two tryptophan residues present in the three-dimensional (3D) structure of the protein at positions Trp189 and Trp389, respectively. This result indicates that the loss of catalytic activity in the presence of Gdn-HCl is due to alterations in the overall structure of HpG6PD. When the enzyme is exposed to Gdn-HCl, the fluorescence intensity decreases, compared to the concentration of Gdn-HCl; we observed a fluorescence reduction by 400 a.u. at the highest concentration of Gdn-HCl (2.0 M). It is interesting to note that at 0.4 M of Gdn-HCl, when the HpG6PD enzyme ceased all activity, the intrinsic fluorescence intensity remained the same as when in the absence of Gdn-HCl, indicating that the loss of catalytic activity in the presence of Gdn-HCl is not due to alterations in the overall structure of HpG6PD.

Using these different biophysical experiments allows us to approach a study of the protein from different perspectives. The assays of temperature, proteases, and chaotropic agents (Gdn-HCl) are used to establish the enzyme’s structure and functionality. At the same time, circular dichroism (CD) measures the secondary structure. By using the fluorescence of tryptophan and activity assays, the solvent exposure of one type of amino acid and the requirements for activity (intact active site) were determined. Finally, thermal stability (*T*_m_) measured the global stability of the protein at various temperatures.

Finally, it is important to mention that since HpG6PD is a key enzyme in the metabolism of *H. pylori*, it was of interest to characterize it for its biochemical and catalytic properties; this represents an important initial step in the search for new drugs for *H. pylori*. Thus, the structural and functional characterization of target proteins is an essential step in drug development. It allows the subsequent characterization of the potential effect of compounds to generate new and more selective, effective, and efficient drugs. Furthermore, in the future, we will be able to determine the inhibition type (via the determination of kinetic parameters) of the chemical compounds previously reported for the HpG6PD throughout the in silico assays. In addition, knowledge of the secondary and 3D structure and the oligomeric composition of the native status of HpG6PD ensures that the enzyme HpG6PD will be evaluated in the presence of chemical compounds, to determine the alterations to the protein at secondary, tertiary, and quaternary structure levels.

In addition, through bioinformatic methods, we have been able to predict the 3D structure of HpG6PD and, thus, make a comparison with the homologous enzyme in humans (HsG6PD); the results seem to be very promising, since they present a low percentage of similarity (27%), in addition to a value above 1 for the RMSD parameter (1.423 Å) and a low Q-score value (0.648). This indicates that they show structural differences; this is an advantage when considering the enzyme HpG6PD as a potential pharmacological target.

## 4. Conclusions

We reported the expression and purification of HpG6PD, which allowed us to characterize it as a functional enzyme that participates in the metabolism of glucose in *Helicobacter pylori*. HpG6PD seems to have similar conserved domains that are related to ligand binding. The recombinant HpG6PD protein has a molecular mass of 54 kDa and the native state of the protein in solution is a catalytic dimer. In addition, we determined the kinetic parameters of the HpG6PD protein for both G6P and NADP+ substrates, observing a low catalytic constant compared to G6PDs from other organisms. Furthermore, we found that the thermal stability of HpG6PD was not improved at different concentrations of NADP^+^. However, NADP^+^ protects the HpG6PD from trypsin digestion and Gdn-HCl, contrary to G6P. Our current results suggest that we could determine the mechanisms of action of synthetic compounds using the activity of HpG6PD and propose it as a potential therapeutic target.

## Figures and Tables

**Table 1:** Previously steady-state kinetic parameters, as reported for G6PDs enzymes.

Organism	*k_cat_* (s^−1^)	*K_m_* G6P (µM)	*K_m_* NADP^+^ (µM)	Reference
*Helicobacter pylori*	70	75	12	In this study
*Gluconacetobacter diazotrophichus*	293,181	63	7	[34]
*Escherichia coli* DH5α	32	224	127	[50]
*Pseudomonas aeruginosa*	540	498	56	[44]
*Termotoga maritima*	35,000	200	40	[43]
*Haloferax volcanii*	11	370	520	[44]
*Giardia lamblia*	31	18	14	[53]
*Plasmodium falciparum*	8	19	6	[54]
*Trypanosoma cruzy*	62	77	16	[52]
*Aspergillus niger*	NR	153	26	[55]
*Aspergillus oryzae*	1000	109	6	[56]
*Brugia malayi*	40	245	14	[49]
Dog liver	NR	122	10	[57]
Buffalo liver	NR	NR	59	[48]
Camel liver	NR	81	81	[47]
*Homo sapiens*	230	38	7	[32]

## Supplementary Materials

The following supporting information can be downloaded at: https://www.mdpi.com/article/10.3390/microorganisms10071359/s1, Figure S1. Schematic presentation of the pET3aHisTEVP-*zwf* vector used for the production of G6PD protein. Figure S2. Structural alignment of the HpG6PD model with HsG6PD. Figure S3: Multiple-sequence alignment of the G6PDs amino acid sequences.
