# Supplementary material for: Biochemical and Kinetic Characterization of the Glucose-6-Phosphate Dehydrogenase from Helicobacter pylori Strain 29CaP
Source: Microorganisms. 2022 Jul 6;10(7):1359. doi: 10.3390/microorganisms10071359 (PMC9323780; doi:10.3390/microorganisms10071359)
Supplement: Supplementary file 1 [file microorganisms-10-01359-s001.zip › microorganisms-1795032-supplementary.pdf]

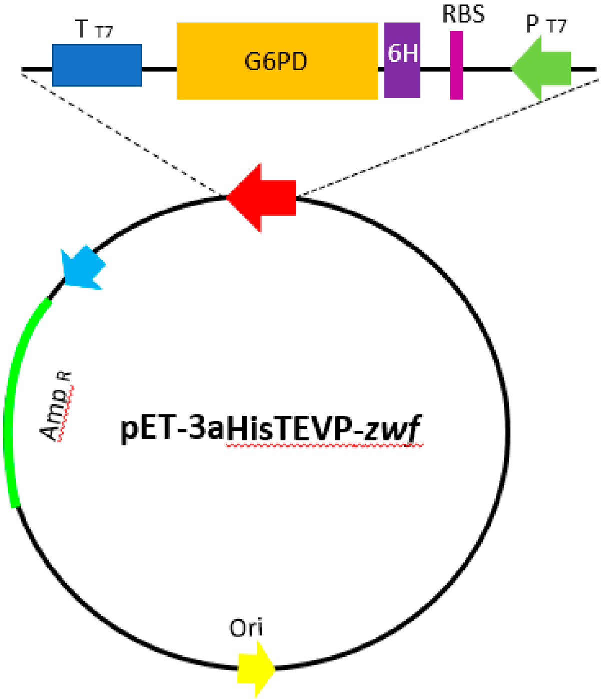

Figure S1. Schematic presentation of the pET3aHisTEVP-*zwf* vector used for the production of G6PD protein. pET3aHisTEVP-*zwf* vector carrying the *zwf* gene from *Helicobacter pylori* and regulated by a T7 promoter.

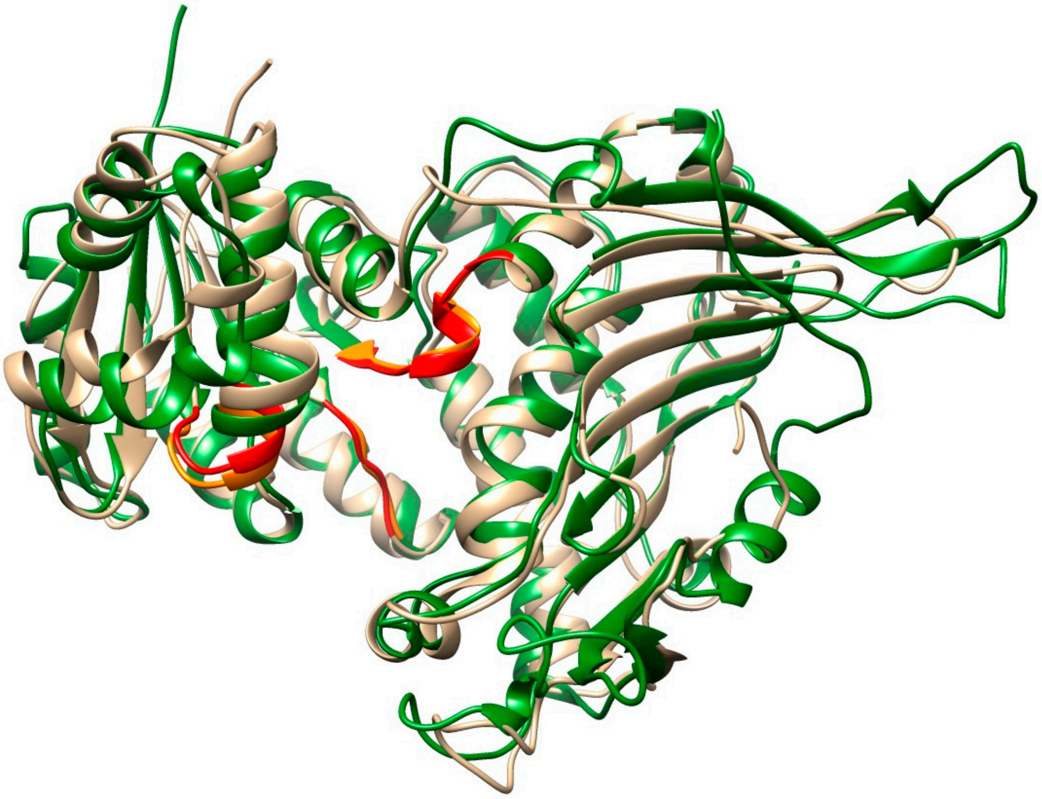

Figure S2. Structural alignment of HpG6PD model with HsG6PD. The structural alignment was performed using Chimera 1.14, the HpG6PD is shown in light brown, and HsG6PD in shown in green; the catalytic site is in red.

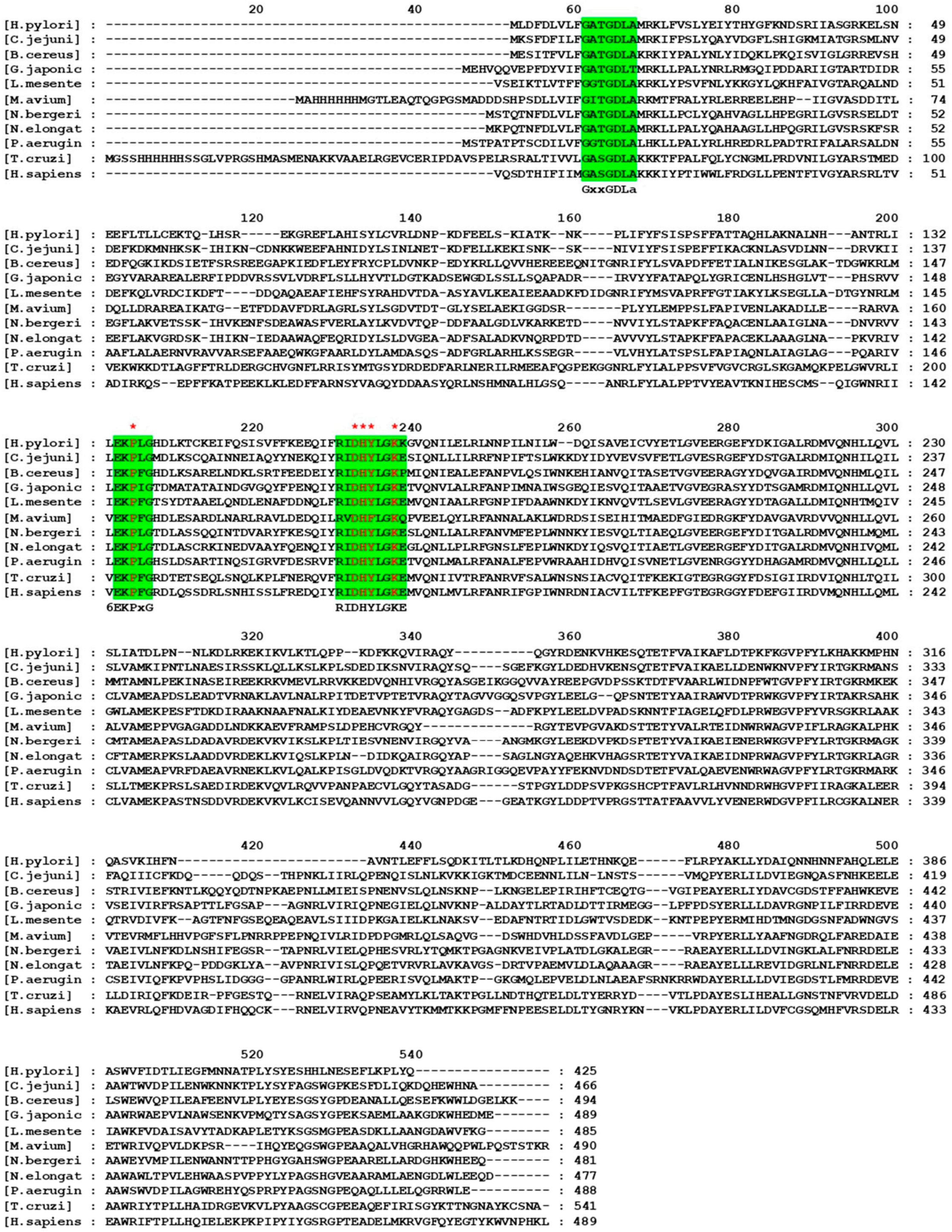

Figure S3. Multiple sequences alignment of G6PDs amino acids sequences. The sequences of G6PDs used for alignment were ALM79592.1 (*Helicobacter pylori*), WP_126241084.1 (*Campylobacter jejuni*) WP_098253162.1 (*Bacillus cereus*), WP_061933270.1 (*Gluconobacter japonicus*), 1DPG (*Leuconostoc mesenteroides),* 4LGV (*Mycobacterium avium*), WP_118792099.1 (*Neisseria bergeri*), WP_204813281.1 (*Neisseria elongate*), WP_024917865.1 (*Pseudomonas aeruginosa*), 6D23 (*Trypanosoma cruzi*), 6VA8 (*Homo sapiens*).
